# Prediction of ovarian function in premenopausal breast cancer patients with amenorrhoea after chemotherapy: a simple clinical score

**DOI:** 10.1186/s40064-016-2671-x

**Published:** 2016-07-11

**Authors:** Xiao Shi Li, Qing Lv, Zheng Gui Du, Jie Chen

**Affiliations:** Department of Thyroid and Breast Surgery, West China Hospital of Sichuan University, Guo xue Lane 37, Chengdu, China

**Keywords:** Breast cancer, Ovarian function, Amenorrhoea, Ovariectomy, Sex hormone

## Abstract

We evaluated a non-invasive method for predicting the ovarian function of premenopausal breast cancer patients with amenorrhoea after chemotherapy. A total of 34 patients had ovarian function, whereas 56 had no ovarian function. Logistic regression analysis indicated that age (*P* = 0.034; hazards ratio [HR], 0.29; confidence interval [CI], 0.091–0.910), follicle-stimulating hormone (*P* = 0.032; HR 0.97; CI 0.944–0.997) and oestradiol (*P* = 0.047; HR 1.01; CI 1.000–1.015) were independent influencing factors that determine ovarian function. The ovarian function score (OFS) (*P* < 0.001; HR 48.00; CI 10.174–226.452) was obtained through a comprehensive analysis of these three variables, and it could more effectively predict ovarian function. According to receiver operating characteristic curve analysis, the OFS had the highest values compared with the other three variables (sensitivity, 94.6 %; specificity, 79.3 %). The OFS is simple and easy to use; thus, it is expected to become a new method for determining drug-induced amenorrhoea in women with breast cancer. Ovarian function likely still exists if the OFS is ≤1.

## Background

Endocrine therapy is one of the important systemic treatment methods conventionally recommended for patients with breast cancer with a positive hormone receptor, and it can effectively reduce mortality due to hormone-dependent breast cancer and its recurrence rates (Jordan 2014; Davies et al. 2013).

Oestrogen in premenopausal women mainly comes from the ovaries, and its production is affected by gonadotropins. Premenopausal breast cancer patients often choose tamoxifen (TAM), a selective oestrogen receptor modulator, as their first-line endocrine treatment. Clinical studies such as ATAC (Cuzick et al. 2010) and BIG 1-98 (Breast International Group (BIG) 1-98 Collaborative Group et al. 2005) have confirmed that the curative effect of aromatase inhibitors (AIs) in patients with breast cancer is superior to TAM. However, AIs can only block the synthesis of exogenous oestrogen outside the ovaries in women, e.g., by blocking the adrenal cortex or peripheral tissues (i.e., the liver, fat and muscle), to convert testosterone into oestradiol (E2) and androstenedione into oestrone. Thus, AIs can only be used for patients with breast cancer with ovarian function failure. Prediction of ovarian function in premenopausal breast cancer patients with amenorrhoea after chemotherapy is important.

Periodic monitoring detection of serum sex hormone levels is often used to clinically evaluate ovarian function, as serum E2 and follicle-stimulating hormone (FSH) levels are used in evaluating menopausal status (Yu et al. 2010). About 10 % of patients with amenorrhoea resume bleeding 2 years after chemotherapy (Sukumvanich et al. 2010). Meanwhile, TAM can influence the ovarian function of premenopausal patients, with several clinical trials confirming that it may induce amenorrhoea (Tham et al. 2007; Ganz et al. 2011; Jung et al. 2010). Researchers have found that AIs should be avoided in patients with amenorrhoea <48 years who are receiving TAM, despite menopausal manifestations and sex hormone level changes (Guerrero et al. 2013). About 25–30 % of amenorrheic breast cancer patients’ ovarian function may be misdiagnosed when determined through their menopausal status and sexual hormones (Smith et al. 2006; Henry et al. 2013). This suggests that it is very difficult to determine ovarian function accurately.

Ovarian pathology results are the gold standard, as they accurately reflect ovarian functions. In this retrospective study, we collected clinical and pathological data of patients with breast cancer who developed amenorrhoea after chemotherapy and underwent ovariectomy for their condition. We observed the largest sectional area of the ovary and determined whether it had function by examining the ovarian pathology results. The relationship between the clinical follow-up data before ovariectomy and the ovarian function was tested. The present study attempted to determine more reliable clinical indexes and a non-invasive method for evaluating ovarian function.

## Methods

Data of patients initially diagnosed with breast cancer in West China hospital of Sichuan University during 2008 to 2013 were collected. Patients with normal menstruation before breast cancer treatment, with premenopausal level of sexual hormones (hormones are normal before chemotherapy: FSH ≤ 40 IU/L, E2 ≥ 10 pg/mL) and with cessation of menstruation during chemotherapy; those with a positive hormone receptor (positive for oestrogen, progesterone, or both); and those treated with TAM after completing chemotherapy were included. Patients who used endocrine therapy drugs other than TAM; those who used drugs, within 6 months, that may interfere with sexual hormone levels; and those who had already undergone hysterectomy were excluded.

Castration was performed for the following reasons: (Jordan 2014) breast cancer accompanied with distant metastasis during initial diagnosis; (Davies et al. 2013) high recurrence risk with amenorrhoea after chemotherapy and menstruation resumption during follow-up; (Cuzick et al. 2010) recurrence or metastasis during the treatment process. All patients took TAM for less than 5 years, and none desired fertility preservation. After a discussion with their doctors, the patients opted for ovarian castration combined with AI treatment for better therapeutic effect. Because they refused to use drug castration due to financial burden or personal reasons (e.g., they could not receive long-term injections), they voluntarily chose to undergo oophorectomy.

## Data collection

The following clinical data were collected from electronic medical records and follow-up databases of West China hospital of Sichuan University (follow-up once every 3 months in the first year after breast cancer surgery and once every 6 months after the first year): age, body mass index, number of pregnancies, number of births, menarche age, tumour size, lymph node stage, hormone receptor status, immunochemical human epidermal growth factor receptor 2 (her-2) score, targeted therapy (herceptin, 8 mg/kg for the first time, 6 mg/kg afterwards for maintenance every 3 weeks), chemotherapy cycle, chemotherapy regimen, duration of TAM administration, duration between completing chemotherapy and ovariectomy, ovarian cyst and its nature (e.g., metastatic tumour of breast cancer; benign epithelial tumour; functional cysts, including follicular cysts and corpus luteum cysts), histological grade and the date when bleeding resumed. The following chemotherapy regimens were used: without anthracycline and cyclophosphamide: taxanes (175 mg/m^2^) every 3 weeks; with either anthracycline or cyclophosphamide: taxotere (75 mg/m^2^) + cyclophosphamide (600 mg/m^2^) every 3 weeks; epirubicin (100 mg/m^2^) + taxotere (75 mg/m^2^) every 3 weeks; with both anthracycline and cyclophosphamide: epirubicin (100 mg/m^2^) + cyclophosphamide (830 mg/m^2^) every 3 weeks; taxotere (75 mg/m^2^) + epirubicin (100 mg/m^2^) + cyclophosphamide (500 mg/m^2^) every 3 weeks, fluorouracil (500 mg/m^2^) + epirubicin (100 mg/m^2^) + cyclophosphamide (500 mg/m^2^) every 3 weeks, followed by taxotere (100 mg/m^2^) every 3 weeks. The dosage of TAM was 20 mg/day.

The FSH, luteinising hormone (LH) and E2 levels were assessed at each follow-up. If bleeding resumed in patients who had amenorrhoea after chemotherapy, the sexual hormone levels were reassessed on the second day of each menstrual cycle. The sex hormone levels were determined by chemiluminescence immunoassay (Roche E170; Roche Diagnostics, Mannheim, Germany), which defined postmenopausal hormone levels as an FSH > 26.1 IU/L; LH > 14.4 IU/L; E2 < 5 pg/mL. Ovarian tissue was divided equally into two parts, and the pathological sections were assessed independently by two experienced pathologists. The ovary were determined to have developed ovarian follicles when ovarian pathology confirmed the existence of undegraded corpus luteum, the primary or secondary follicles, follicular cysts, corpus luteum cysts. When there were scattered and atretic follicles and occasional primordial follicles, ovarian function was determined to have decreased without complete failure. The aforementioned situations represent existing ovarian function. The absence of such conditions was identified as ovarian failure (Nichols et al. 2005; Ozdamar et al. 2005).

### Ethics approval and consent to participate

This study was approved by the ethical committee of the West China hospital of Sichuan University.

### Statistical analysis

Univariate analysis was performed to screen the variables that had a significant association with ovarian function. Categorical variables were analysed using Pearson’s χ^2^ test. Quantitative variables were analysed using *t* test. Ranked data were analysed using the Kruskal–Wallis test. If *P* < 0.1, the variable was used in logistic regression analysis to analyse the independent factors to determine ovarian function. Receiver operating characteristic (ROC) curves were used to describe the optimal prediction thresholds of the variables, and the area under the curve (AUC) indicated a factor’s predictive value for determining ovarian function. After performing multivariate analysis, the ROC curve was used to establish a simpler and easier scoring method.

## Results

The clinical characteristics of these participants are shown in Table 1. Ninety patients were included. Thirty-four patients had ovarian function, whereas 56 had no ovarian function. Variables with *P* < 0.1, as confirmed by univariate analysis, are shown in Table 1, including age (≥40 or <40 years, *P* = 0.002), tumour size (*P* = 0.013), lymph node stage (*P* = 0.023), duration of TAM (*P* = 0.035), duration between completing chemotherapy and ovariectomy (*P* = 0.046), LH (*P* < 0.001), FSH (*P* < 0.001), E2 (*P* = 0.002) and ovarian cyst (*P* < 0.001).

**Table 1:** Participants’ clinical characteristics

Characteristic	With ovarian function (n = 34)	Without ovarian function (n = 56)	P value
Age [% (years)]			0.002
≥40 (n = 67)	19 (28.4)	48 (71.6)	
<40 (n = 23)	15 (65.2)	8 (34.8)	
BMI (kg/m^2^, mean ± SD)			0.136
<24 (n = 65)	21 (32.3)	44 (67.7)	
24–28 (n = 20)	12 (60.0)	8 (40.0)	
≥28 (n = 5)	1 (20)	4 (80)	
Menarche age (years, mean ± SD)	13.6 ± 1.5	13.8 ± 1.5	0.692
No. of pregnancies (mean ± SD)	2.7 ± 1.5	2.8 ± 1.4	0.918
No. of births (mean ± SD)	1.1 ± 0.3	1.2 ± 0.6	0.414
Pathological classification (%)			0.450
Ductal carcinoma (n = 82)	31 (37.8)	51 (62.2)	
Adenocarcinoma (n = 2)	0 (0)	2 (100.0)	
Lobular carcinoma (n = 6)	3 (50.0)	3 (50.0)	
Histological classification (%)			0.160
1 (n = 2)	0 (0)	2 (100.0)	
2 (n = 35)	11 (31.4)	24 (68.6)	
3 (n = 53)	23 (43.4)	30 (56.6)	
Tumor size			0.013
T1 (n = 28)	15 (53.6)	13 (46.4)	
T2 (n = 52)	18 (34.6)	34 (56.4)	
T3 (n = 10)	1 (10.0)	9 (90.0)	
Lymph node stage			0.023
N0 (n = 20)	9 (45.0)	11 (55.0)	
N1 (n = 26)	15 (57.7)	11 (42.3)	
N2 (n = 18)	4 (22.2)	14 (77.8)	
N3 (n = 26)	6 (23.1)	20 (76.9)	
ER status			0.839
ER+ (n = 84)	31 (36.9)	53 (63.1)	
ER− (n = 6)	3 (50.0)	3 (50.0)	
PR status			1.000
PR+ (n = 78)	29 (37.2)	49 (62.8)	
PR− (n = 12)	5 (41.7)	7 (58.3)	
HER-2 score (%)			0.515
0 (n = 59)	21 (35.6)	38 (64.4)	
1 (n = 10)	5 (50.0)	5 (50.0)	
2 (n = 13)	3 (23.1)	10 (76.9)	
3 (n = 8)	5 (62.5)	3 (37.5)	
Targeted therapy (%)	3 (33.3)	6 (66.7)	1.000
Chemotherapy regimens (%)			0.734
Taxanes (n = 1)	0 (0)	1 (100.0)	
Anthracycline or cyclophosphamide (n = 31)	12 (38.7)	19 (61.3)	
Both anthracycline and cyclophosphamide (n = 58)	22 (37.9)	36 (62.1)	
Chemotherapy cycle (mean ± SD)	6.5 ± 1.2	6.5 ± 1.3	0.676
Duration of tamoxifen (month, mean ± SD)	14.0 ± 13.0	9.9 ± 12.6	0.035
Duration between completing chemotherapy and ovariectomy (month, mean ± SD)	14.7 ± 12.7	10.7 ± 12.2	0.046
Ovarian cyst (%)	15 (44.1)	6 (10.7)	<0.001
Metastatic tumor of breast cancer (n = 1)	1 (100)	0 (0)	
Benign epithelial tumor (n = 10)	4 (40)	6 (60)	
Functional cysts (n = 10)	10 (100)	0 (0)	
FSH (IU/L, mean ± SD)	26.0 ± 2.0	45.2 ± 23.9	<0.001
LH (IU/L, mean ± SD)	15.2 ± 10.2	24.2 ± 12.1	<0.001
E2 (pg/ml, mean ± SD)	156.3 ± 311.7	19.6 ± 57.4	0.002

**Fig. 1:**
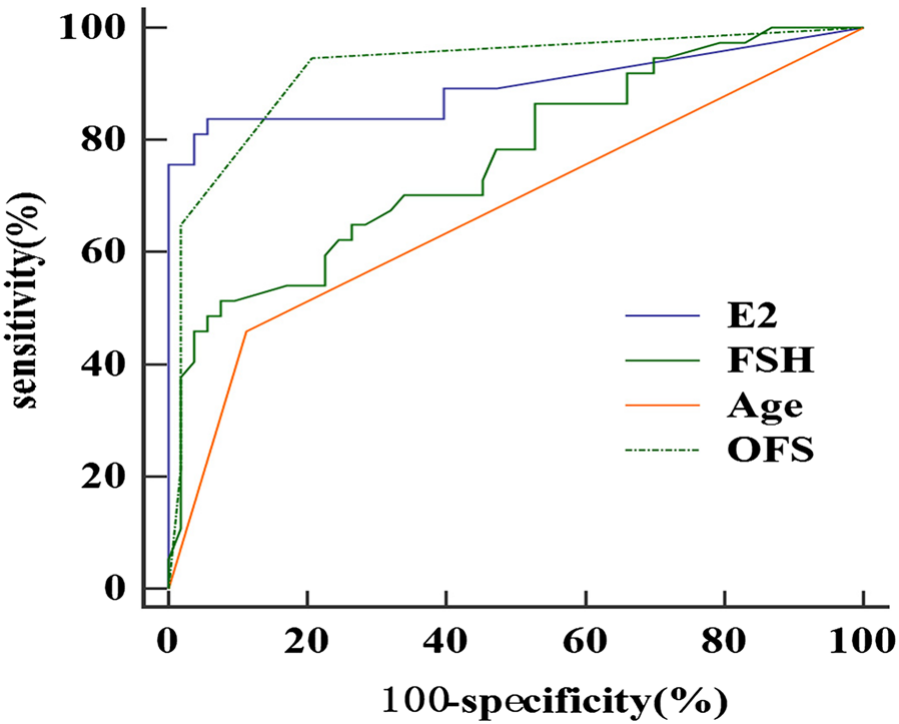
The ROC curve compare between estradiol, Follicle-Stimulating hormone, age, and ovarian function score

After including the aforementioned variables, age (*P* = 0.034; hazards ratio [HR], 0.29; confidence interval [CI] 0.091–0.910), FSH (*P* = 0.032; HR 0.97; CI 0.944–0.997) and E2 (*P* = 0.047; HR 1.01; CI 1.000–1.015) were independent influencing factors that could determine ovarian function (Table 2). The risk of ovarian failure increased when the patient’s age was ≥40 years, the FSH level increased, and the E2 level decreased. ROC analysis showed that FSH ≤ 23.8 IU/L and E2 > 13.5 pg/mL are the maximum efficiency cut-off levels for a functional ovary. The ovarian function score (OFS) was created, with points assigned to different variables: age, ≥40 years = 1, <40 years = 0; FSH, >23.8 IU/L = 1, ≤23.8 IU/L = 0; E2, >13.5 pg/mL = 1, ≤13.5 pg/mL = 0; OFS = age + FSH-E2. Logistic regression analysis indicated that this new variable was associated with ovarian function (*P* < 0.001; HR 48.00; CI 10.174–226.452), and the ROC curve was generated. The OFS had the largest AUC (0.924) compared with the AUCs of the other four variables and was more discriminating than age (*P* < 0.001) and FSH level (*P* = 0.001). ROC analysis indicated that OFS ≤ 1 is the maximum efficiency score for predicting ovarian function (sensitivity, 94.59 %; specificity, 79.25 %) (Table 3; Fig. 1). Table 4 shows the status of age, FSH level, and E2 level when the ovaries were functional.

**Table 2:** Evaluation of the association between the variables and ovarian function

	HR	95 % CI	P
Age	0.29	0.091–0.910	0.034
FSH	0.97	0.944–0.997	0.032
E2	1.01	1.000–1.015	0.047
OFS	48.00	10.174–226.452	<0.001

**Table 3:** Predictive value of each variable according to the receiver operating characteristic curve analysis

	AUC	Sn (%)	Sp (%)	P
Age	0.673	45.9	88.7	0.001
FSH	0.764	51.4	92.5	<0.001
E2	0.895	83.8	94.3	0.47
OFS	0.924	94.6	79.3	–

P value represent the significance between age/FSH/E2 and OFS

**Table 4:** The status of age, the follicle-stimulating hormone and estradiol levels when the ovaries are functional

OFS	Status of variables (age years; FSH IU/L; E2 pg/mL)
−1	Age < 40 years, FSH ≤ 23.8 and E2 > 13.5
0	Age < 40 years, FSH > 23.8, E2 > 13.5; aged ≥ 40 years, FSH ≤ 23.8, E2 > 13.5; age < 40 years, FSH ≤ 23.8, E2 ≤ 13.5
1	Age < 40 years, FSH > 23.8, E2 ≤ 13.5; age ≥ 40 years, FSH ≤ 23.8, E2 ≤ 13.5; age ≥ 40 years, FSH > 23.8, E2 ≥ 13.5

## Discussion

According to the National Cancer Comprehensive Network, the criteria for determining postmenopausal breast cancer include prior bilateral oophorectomy, >60, <60 years and amenorrhoea for ≥12 months in the absence of chemotherapy, TAM, toremifene, or ovarian suppression, and postmenopausal range of FSH and E2 levels; if a patient <60 years is taking TAM or toremifene, the FSH and E2 levels should be in the postmenopausal range (National Comprehensive Cancer Network 2013). AI is foreseen to be prescribed as serial hormone measurements showed FSH > 40 UI/L and E2 < 10 pg/mL (Torino et al. 2014). However, for patients who use TAM, the accuracy of this method is limited and it cannot predict the state of ovarian function. Unfortunately, until now, ovarian function failure could not be accurately determined in menopausal patients with breast cancer after chemotherapy.

The ovarian function begins to decrease starting 40 years old. Most women >40 years develop chemotherapy-induced amenorrhoea, but in patients <40 years, amenorrhoea lasts >6 months after chemotherapy and menstruation recovery often occurs during the follow-up period (Walshe et al. 2006; Minisini et al. 2009). Multiple studies have suggested that the risk of ovarian failure after chemotherapy increases with age (Sonmezer and Oktay 2006; Marhhom and Cohen 2007). The incidence of chemotherapy-induced menopause in patients <40 years (22–61 %) is lower than that in those >40 years (61–97 %) (Del Mastro et al. 1997). The patients in our study were grouped by this cut-off age, and we found that age is an independent factor of ovarian function; in those >40 years, ovarian function decreased more than those <40 years (28.4 vs. 65.2 %). TAM could induce the negative feedback on pituitary gonadotropin because of its oestrogen-like effect (Jordan et al. 1987). In patients who used TAM in our study, this may be because the drug interferes with sexual hormones and thus induces a decrease in the FSH level (Rossi et al. 2009; Harper-Wynne et al. 2002). An FSH level >23.8 IU/L in the OFS is the threshold used to determine non-functional ovaries and slightly lower than the recommended FSH reference value of >26.1 IU/L, according to the detection method used in this study.

Clinical studies have found that TAM can increase the E2 level, which may be due to the direct effect of TAM on ovarian granulosa cells (Metindir et al. 2005; Groom and Griffiths 1976). Mahran et al. (2013) tested female Sprague–Dawley rats and found that radiation inhibited the rats’ ovarian function and oral TAM improved the insulin-like growth factor 1 levels of the follicles. Insulin-like growth factor 1 acts on its receptors, promotes the proliferation of granulosa cells, and maintains the effects of aromatase, which may further promote follicle development. It is possible that this is one of the mechanisms of ovarian hyperstimulation by TAM. Most studies have reported that TAM increases the risk of ovarian cyst in patients with breast cancer, and these cysts typically develop 3–11 months after undergoing treatment and are very rare after 2 years (Metindir et al. 2005; Partridge et al. 2003; Mofrad et al. 2010). TAM-induced ovarian cysts are usually functional ovarian cysts (physiological ovarian cysts) that are formed by the abnormal accumulation of liquid in the follicle or corpus luteum, which is followed by the formation of follicular cysts or corpus luteum cysts (Cohen et al. 1999). In the present study, 17 patients (50 %) with ovarian function had an ovarian cyst, including 13 (76.4 %) functional cysts, with cyst diameters >5 cm in 2 cases. Additionally, patients with an ovarian cyst usually had an increased oestrogen level as high as 1550 pg/mL. The increase in the E2 level and ovarian cysts may be due to ovarian hyperstimulation caused by TAM (Madeddu et al. 2014). In all the patients who used TAM, an E2 level ≥13.5 pg/mL in the OFS predicts ovarian function, and this value is slightly higher than the standard mentioned above.

In our study, age, FSH level and E2 levels were associated with ovarian function, but the accuracy of predicting ovarian function is limited by one of these indexes, as it is easy to misjudge ovarian function. The OFS consists of the three aforementioned clinical data. OFS ≤ 1 can effectively predict ovarian function (sensitivity, 94.59 %; specificity, 79.25 %). Our study findings suggest that comprehensively combining these three variables greatly improves the accuracy of the prediction. The AUC of the OFS (0.924) in ROC analysis was greater than that of age (0.673), FSH (0.764), and E2 (0.895), indicating that it has the greatest prediction value.

Interestingly, we found that the corpus luteum was still visible at 6–12 months in the ovarian tissue of patients with amenorrhoea who were receiving TAM. These findings indicate that these patients may retain ovarian function, and they may experience irregular ovulation or follicles that do not rupture (because of the relatively low LH levels) or are directly luteinised. Therefore, it is possible that TAM prolongs the life of the corpus luteum. For example, Swahn et al. (1989) studied the role of TAM on the corpus luteum of 16 women with normal ovarian function and found that their luteal phase was prolonged compared with that of the control patients. Furthermore, patients who received TAM exhibited elevated sex hormone levels (e.g., FSH, oestrogen and 17-hydroxy-progesterone levels). Moreover, Cirpan et al. (2008) tested the effects of TAM in rats and found that it did not increase the levels of Ki-67 (a cell proliferation marker) in the rats’ corpus luteum versus those of the control group. Therefore, this pathway may affect the extension of the luteal phase. The corpus luteum is the main source of progesterone; however, we did not evaluate this hormone in this study. Therefore, future studies should examine the effect of TAM on progesterone in patients with amenorrhoea. Those data may be useful in determining whether progesterone can be used to predict ovulation and restore ovarian function.

The most accurate method for determining ovarian function is through the pathological examination of serial sections of the entire ovary, which is labour-intensive and cannot be performed in clinical practice. We examined the largest ovarian area possible using consistent sampling methods, yielding reliable results. Currently, for patients with cessation of menstruation after chemotherapy, periodic monitoring of sex hormones cannot accurately predict ovarian function, especially in those who use TAM because it significantly changes sex hormone levels, making it difficult to assess ovarian function. The OFS is a simple, effective and feasible method for evaluating ovarian function. However, our study’s sample size was limited. In the future, multi-centre studies with larger sample sizes should be conducted to further confirm and improve the OFS and provide a basis for future clinical assessments of ovarian function of breast cancer patients.

## Conclusion

The OFS is expected to become a new simple and effective method for determining the ovarian function of women with breast cancer who have drug-induced amenorrhoea.

## Abbreviations

AI: aromatase inhibitor
AUC: area under the curve
CI: confidence interval
E2: oestradiol
FSH: follicle-stimulating hormone
HR: hazards ratio
LH: luteinising hormone
OFS: ovarian function score
ROC: receiver operating characteristic
TAM: tamoxifen
